# Associations of macular microvascular parameters with cerebral small vessel disease in rural older adults: A population-based OCT angiography study

**DOI:** 10.3389/fneur.2023.1133819

**Published:** 2023-03-16

**Authors:** Zhe Xu, Yi Dong, Yongxiang Wang, Lin Song, Sijie Niu, Shanshan Wang, Mingqing Zhao, Jiafeng Wang, Lin Cong, Xiaojuan Han, Tingting Hou, Shi Tang, Qinghua Zhang, Yifeng Du, Chengxuan Qiu

**Affiliations:** ^1^Department of Neurology, Shandong Provincial Hospital, Shandong University, Jinan, Shandong, China; ^2^Department of Neurology, Shandong Provincial Hospital Affiliated to Shandong First Medical University, Jinan, Shandong, China; ^3^Shandong Provincial Clinical Research Center for Neurological Diseases, Jinan, Shandong, China; ^4^Shandong Provincial Key Laboratory of Network Based Intelligent Computing, School of Information Science and Engineering, University of Jinan, Jinan, Shandong, China; ^5^Aging Research Center and Center for Alzheimer Research, Department of Neurobiology, Care Sciences and Society, Karolinska Institutet-Stockholm University, Stockholm, Sweden

**Keywords:** macular microvascular signs, cerebral small vessel disease, optical coherence tomography angiography, magnetic resonance imaging, population-based study

## Abstract

**Objective:**

To explore the associations of macular microvascular parameters with cerebral small vessel disease (CSVD) in rural-dwelling older adults in China.

**Methods:**

This population-based cross-sectional study included 195 participants (age ≥ 60 years; 57.4% women) in the optical coherence tomographic angiography (OCTA) sub-study within the Multimodal Interventions to delay Dementia and disability in rural China (MIND-China). Macular microvascular parameters were measured using the OCTA. We automatically estimated volumes of gray matter, white matter, and white matter hyperintensity (WMH), and manually assessed numbers of enlarged perivascular spaces (EPVS) and lacunes on brain magnetic resonance imaging. Data were analyzed with the general linear models.

**Results:**

Adjusting for multiple confounders, lower vessel skeleton density (VSD) and higher vessel diameter index (VDI) were significantly associated with larger WMH volume (*P* < 0.05). Lower VSD and foveal density-300 (FD-300) of left eye were significantly associated with lower brain parenchymal volume (*P* < 0.05). In addition, lower areas of foveal avascular zone (FAZ) and FD-300 of left eye were significantly associated with more EPVS (*P* < 0.05). The associations of abnormal macular microvascular parameters with WMH volume were evident mainly among females. Macular microvascular parameters were not associated with lacunes.

**Conclusion:**

Macular microvascular signs are associated with WMH, brain parenchymal volume, and EPVS in older adults. The OCTA-assessed macular microvascular parameters can be valuable markers for microvascular lesions in the brain.

## Introduction

Cerebral small vessel disease (CSVD) refers to a group of pathological processes with various etiologies affecting the small arteries, venules, and capillaries of the brain (1). Previous studies have shown that CSVD is associated with clinical stroke and cognitive impairment (2). Therefore, it is important to identify simple, non-invasive, and inexpensive markers for CSVD.

Microvasculatures in the brain and retina are similar in embryologic origin, anatomic features, metabolic activities, patterns of vascularization, and extracellular deposits (3). Thus, the retinal microvasculature could be a potential vehicle for studying changes in the cerebral vasculature. Some population-based studies have examined the associations of alterations in retinal microvasculature and imaging markers of CSVD, in which retinal microvascular signs (e.g., retinal focal arteriolar narrowing and arteriovenous nicking) were assessed using fundus photography (4, 5). However, due to limited resolution and sensitivity, traditional fundus imaging techniques can only qualitatively measure signs of microangiopathy (e.g., micro-hemorrhages and micro-aneurysms) or quantitatively measure arteriolar and venular parameters, but cannot directly quantify parameters of the capillary system (e.g., vessel density and vessel diameter index) (6). As a non-invasive and label-free technique, optical coherence tomography angiography (OCTA) can quantitatively detect the movement of red blood cells at capillary-level resolution and offer imaging markers for retinal microvascular signs, such as retinal vessel density (VD), foveal avascular zone (FAZ), and vessel diameter index (VDI) (6). Previous studies showed that VD was related to the CSVD burden or global white matter hyperintensity (WMH) (7, 8). However, most of these studies have been conducted in the clinical settings of patients with mild cognitive impairment or Alzheimer's disease (AD), and data from the general population settings are sparse. In addition, cerebral WMH is typically located in paraventricular and deep white matter regions, and etiopathological mechanisms of WMH differ depending on their location (9). However, the potential differential associations of macular microvascular parameters with paraventricular and deep WMH have rarely been explored. Furthermore, the sex differences in the associations of retinal microvascular signs with CSVD in older adults remain to be clarified. This is important because sex differences in CSVD and retinal capillary plexus have been frequently reported (10, 11) and there are substantial sex differences in the prevalence of lifestyle risk factors (e.g., smoking and alcohol consumption), especially among rural older adults in China (12, 13).

Thus, in this population-based study, we aimed to investigate the associations of macular microvascular signs with markers of CSVD among dementia-free older adults who were living in the rural communities in China. We hypothesize that macular microvascular signs are associated with CSVD markers and that the associations may vary by sex.

## Methods

### Study design and participants

This population-based cross-sectional study used data from the baseline survey of the Multimodal Interventions to delay Dementia and disability in rural China (MIND-China), which is part of the World-Wide FINGERS Network, as previously reported (14). In brief, baseline assessments of MIND-China targeted people who were aged ≥ 60 years by the end of 2017 and living in the 52 villages of Yanlou Town, Yanggu County, western Shandong Province, China. In March–September 2018, 5,765 participants (57.2% female) were examined during the baseline survey of MIND-China (15).

From June 2019 to November 2020, 284 participants accomplished the brain MRI and OCTA examination in Southwest Lu Hospital. The cluster (village)-randomized sampling approach was used in the selection of participants for the MRI and OCTA substudies in MIND-China. Of these, 47 were excluded due to missing data on OCTA images of left eye (*n* = 30) or right eye (*n* = 17). We further excluded 42 participants due to prevalent dementia (*n* = 2), and suboptimal quality of OCTA images (*n* = 31) and brain MRI images (*n* = 9). Therefore, the final analytical sample included 195 dementia-free participants. Compared to the MIND-China participants who were not included in this study (*n* = 5,570), those in the analytical sample (*n* = 195) were slightly younger (mean age 67.95 vs. 70.99 years, *P* < 0.001) and more educated (mean years of schooling education 3.66 vs. 3.16 years, *P* = 0.041), but the two groups did not differ significantly in the distribution of sex (female 57.44 vs. 57.18%, *P* = 0.944).

The MIND-China study was approved by the Ethics Committee at Shandong Provincial Hospital. All participants were informed of the study protocol in detail and signed the informed consent form. Research within MIND-China was conducted in accordance with the ethical principles expressed in the Declaration of Helsinki. MIND-China was registered in the Chinese Clinical Trial Registry (Registration No. ChiCTR1800017758).

### Data collection and assessments

In March-September 2018, the trained research staff collected data through face-to-face interviews, clinical examinations, neuropsychological tests, and laboratory tests, as part of the baseline examinations of MIND-China (15). The data included demographic features (e.g., age, sex, and education), lifestyles (e.g., smoking and alcohol consumption), health history (e.g., hypertension, diabetes, and dyslipidemia). Smoking and alcohol intake were classified as never and ever smoking or consuming alcohol. The data collection and assessments were previously described in detail (15).

### MRI acquisition and assessment protocols

Participants were scanned on the Philips Ingenia 3.0T MR System in the Southwestern Lu Hospital. The MRI protocols (acquisition, sequences, processing, and quantification) were previously described in detail (15, 16). The protocol included a sagittal three-dimensional T1-weighted fast field echo sequence, an axial T2-weighted fast field echo sequence, and a sagittal three-dimensional fluid attenuated inversion recovery (FLAIR) sequence.

We used AccuBrain^®^ (BrainNow Medical Technology Ltd., Shenzhen, Guangdong, China) to assess WMH volume, brain parenchymal volume, and total intracranial volume (ICV), as previously reported (17). AccuBrain^®^ segmented the T1-weighted images and quantified the volumes of gray matter, white matter, and cerebrospinal fluid based on the principle of similarity measures. The ICV was estimated as the sum of gray matter, white matter, and cerebrospinal fluid volume. The brain parenchymal volume was estimated as the sum of gray matter and white matter volume.

To acquire WMH volume, AccuBrain^®^ used T2- FLAIR images to calculate the signal contrast between normal brain tissue and WMH, and set the signal threshold to recognize WMH. Based on predefined threshold, WMH was recognized and extracted using T2-FLAIR images. Finally, AccuBrain^®^ refined and localized WMH using the transformed T1-weighted brain structure mask extracted from our study sample.

Perivascular spaces (PVS) were microscopic fluid-filled structures that surround the small penetrating blood vessels in the brain. The number of enlarged perivascular spaces (EPVS) was counted on T2-weighted images according to a validated protocol (18). The EPVS appear linear when imaged parallel to the course of the vessel, and round or ovoid (diameters <3 mm) when imaged perpendicular to the course of the vessel. EPVS of the basal ganglia (BG) and centrum semiovale (CSO) were visually counted by the trained rater (M.Z.) who was unaware of the clinical information, under the supervision of an experienced clinical neurologist (L.S). The rater first reviewed all MRI slices showing BG and CSO regions and then counted the number of EPVS on the slices with the highest number of EPVS (18). Three months after the initial assessment, EPVS were reassessed on MRI images from 30 randomly selected subjects, and the intra-evaluator correlation coefficients for BG-EPVS and CSO-EPVS were 0.88 and 0.83, respectively. The total number of global EPVS (i.e., BG-EPVS plus CSO-EPVS) was used in our analysis.

Lacune was defined as a region of abnormal signal intensity in a vascular distribution, 3–15 mm in size with a cerebrospinal fluid density on the subtraction image (19). Lacunes were counted on FLAIR images by the trained rater (J.W.) who was blind to clinical information, under the supervision of an experienced clinical neurologist (L.S). Three months after the initial assessment, lacunes were reassessed on MRI images of 200 randomly selected subjects from all participants in MIND-China who undertook brain MRI scans, and the intra-rater correlation coefficients for lacunes was 0.838.

### Optical coherence tomography angiography acquisition and assessment

The OCTA examination was conducted by ophthalmologists in Southwestern Lu Hospital using Spectralis HRA+OCT (Software Version 1.10.2.0; Heidelberg Engineering, Inc., Heidelberg, Germany). Macular OCTA images of 6 × 6 mm areas (10° × 10° scan angle that included 512 A-scans × 512 B-scans, no pre-determined automatic real time) centered at the fovea were acquired in a dark room without pupil dilation. We scanned the right eye first and then the left eye. We excluded images of suboptimal quality due to eye movement, decentration, defocus, shadow, Z offset, and segmentation error (20). OCTA images of both eyes were included and analyzed in this study. Because OCTA signals in the deeper layers carry the shadows of blood vessels from the superficial plexus, our analysis only focused on the superficial vascular plexus (extending from the inner limiting membrane to 17 mm above the inner plexiform layer) (21).

All macular OCTA images were processed into a binary image in MATLAB (R2019a, MathWorks, Inc.) using a global threshold, hessian filter, and adaptive threshold. Macular microvascular parameters, including vascular density (e.g., VD, VSD, and FD-300) and vascular morphology (e.g., FAZ and VDI), were calculated. FAZ area was defined as the area of the avascular region in the center of the blood flow image (22). VD was calculated as the ratio of the total image area occupied by the blood flow signal to the total image area in the binary vessel maps (subtract FAZ area) (22). VSD was calculated as the ratio of the length occupied by the blood flow signal to the total area in the skeletonized vessel map (subtract FAZ area) (22). VDI, defined as the average diameter of blood vessels, was calculated as the ratio of the area covered by blood flow signal to the length of vessel skeletonization (23). FD-300 was defined as the foveal vessel density in a 300-μm wide zone around the FAZ (24).

### Statistical analysis

We reported frequencies (%) for categorical variables and mean (SD) for continuous variables. Characteristics of study participants by sex were compared using non-parametric test for continuous variables, and chi-square test for categorical variables. General linear models were used to examine the associations of macular microvascular parameters with WMH volume, number of lacunes, brain parenchymal volume, and EPVS count. WMH volume and numbers of lacunes and EPVS were log-transformed due to the skewed distribution. By simultaneously entering the independent variables and their cross-product term into the same model, we tested the statistical interactions of retinal microvascular parameters with sex on CSVD and when statistically significant interaction was detected, we further performed stratified analyses by sex. We reported the main results from two models: model 1 was adjusted for age, sex, and education, if applicable, for ICV; and model 2 was additionally adjusted for alcohol consumption, smoking status, hypertension, diabetes, and dyslipidemia. IBM SPSS Statistics for Windows, Version 25.0 (IBM Corp., Armonk, NY, USA) was used for all the analyses. A two-tailed *P* < 0.05 was considered to be statistically significant.

## Results

### Characteristics of the study participants

The mean age of the 195 participants in the analytical sample was 67.95 (SD = 4.01) years, and 57.4% were females. Compared with females, male participants were more educated, and more likely to smoke and drink alcohol, but less likely to have dyslipidemia (*P* < 0.05). In addition, male participants had a smaller FAZ area on both eyes and a smaller brain parenchymal volume but a larger ICV volume (*P* < 0.05; Table 1). The two groups did not differ significantly in mean age, FD-300, VD, VSD, VDI (on both eyes), global WMH volume, periventricular WMH (PWMH) volume, deep WMH (DWMH) volume, numbers of EPVS and lacunes, and in the prevalence of diabetes and hypertension (*P* > 0.05).

**Table 1:** Characteristics of the study participants by sex.

**Characteristics**	**Total (*n* = 195)**	**Female (*n* = 112)**	**Male (*n* = 83)**	***P*-value^a^**
Age (years)	67.95 (4.01)	68.03 (4.09)	67.86 (3.91)	0.696
Education (years)	3.66 (3.66)	1.91 (2.68)	6.02 (3.49)	<0.001
Ever smoking, *n* (%)	63 (32.30)	1 (0.90)	62 (74.70)	<0.001
Ever alcohol drinking, *n* (%)	81 (41.50)	9 (8.00)	72 (86.70)	<0.001
Diabetes, *n* (%)	17 (8.70)	13 (11.60)	4 (4.80)	0.097
Dyslipidemia, *n* (%)	45 (23.10)	32 (28.60)	13 (15.70)	0.034
Hypertension, *n* (%)	130 (66.70)	75 (67.00)	55 (66.30)	0.918
**CSVD**				
ICV (ml)	1,431.03 (128.19)	1,369.03 (110.93)	1,514.70 (99.41)	<0.001
Global WMH volume (ml)	7.68 (10.46)	7.21 (11.05)	8.31 (9.63)	0.247
Periventricular WMH volume (ml)	6.38 (9.80)	6.00 (10.50)	6.89 (8.80)	0.163
Deep WMH volume (ml)	1.30 (1.46)	1.21 (1.36)	1.41 (1.58)	0.595
Brain parenchymal volume (L)	1.09 (0.10)	1.04 (0.09)	1.14 (0.08)	<0.001
EPVS	61.48 (29.93)	58.05 (28.27)	66.10 (31.63)	0.088
Lacunes^b^	0.76 (1.93)	0.56 (1.52)	1.03 (2.35)	0.313
**Macular microvascular index**				
FAZ area (mm^2^), OS	0.59 (0.29)	0.64 (0.33)	0.53 (0.20)	0.030
FAZ area (mm^2^), OD	0.58 (0.25)	0.62 (0.25)	0.53 (0.24)	0.004
FD-300 (%), OS	0.40 (0.04)	0.40 (0.04)	0.40 (0.04)	0.650
FD-300 (%), OD	0.40 (0.04)	0.40 (0.04)	0.39 (0.05)	0.281
VD (%), OS	0.43 (0.03)	0.43 (0.03)	0.43 (0.03)	0.411
VD (%), OD	0.43 (0.03)	0.43 (0.03)	0.43 (0.03)	0.536
VSD (%), OS	0.15 (0.01)	0.15 (0.01)	0.15 (0.01)	0.142
VSD (%), OD	0.15 (0.01)	0.15 (0.01)	0.15 (0.01)	0.558
VDI (μm), OS	33.10 (2.51)	33.45 (2.78)	32.62 (2.02)	0.056
VDI (μm), OD	32.96 (2.55)	33.02 (2.74)	32.89 (2.29)	0.920

Data are mean (standard deviation), unless otherwise specified.

OS, left eye; OD, right eye; ICV, intracranial volume; EPVS, enlarged perivascular space; WMH, white matter hyperintensities; CSVD, cerebral small vessel disease; FAZ, foveal avascular zone; FD-300, foveal vessel density in the 300 μm ring; VD, vessel density; VSD, vessel skeleton density; VDI, vessel diameter index.

a P-value is for the test of differences between male and female.

b Data on lacunes were missing in 6 participants.

### Associations of macular microvascular parameters with global cerebral small vessel disease

Adjusting for age, sex, education, and if applicable, for ICV, lower VSD but higher VDI were significantly associated with higher global WMH volume (*P* < 0.05); Lower FD-300 and VSD of the left eye were significantly associated with lower brain parenchymal volume (*P* < 0.05); Lower FD-300 and FAZ areas of the left eye were significantly associated with more EPVS (*P* < 0.05); these associations remained statistically significant even after further controlling for dyslipidemia, diabetes, hypertension, smoking, and alcohol consumption (*P* < 0.05; Table 2). The number of lacunes was not significantly associated with any of the examined macular microvascular parameters (*P* > 0.05; Table 2).

**Table 2:** β coefficient (95% confidence interval) of cerebral small vessel disease associated with macular microvascular parameters (*n* = 195).

**Macular microvascular index**	**Global WMH volume**		**Brain parenchymal volume**		**EPVS**		**Lacunes** ^**b**^	
	**Model 1** ^a^	**Model 2** ^a^	**Model 1** ^a^	**Model 2** ^a^	**Model 1** ^a^	**Model 2** ^a^	**Model 1** ^a^	**Model 2** ^a^
**OS**								
FAZ area (mm^2^)	0.01 (−0.19, 0.20)	0.03 (−0.17, 0.22)	0.00 (−0.01, 0.02)	0.01 (−0.01, 0.02)	–0.14 (–0.25, –0.03)^*^	–0.14 (–0.25, –0.03)^*^	0.00 (−0.13, 0.14)	0.01 (−0.12, 0.15)
FD-300 (%)	−0.55 (−1.84, 0.73)	−0.47 (−1.75, 0.82)	0.09 (0.01, 0.17)^*^	0.08 (0.00, 0.16)^*^	–0.82 (–1.57, –0.07)^*^	–0.76 (–1.51, 0.00)^*^	−0.38 (−8.42, 4.92)	−0.28 (−1.20, 0.63)
VD (%)	−0.04 (−2.09, 2.00)	0.10 (−1.93, 2.13)	0.12 (−0.01, 0.24)	0.11 (−0.02, 0.23)	−0.60 (−1.81, 0.61)	−0.53 (−1.73, 0.67)	0.08 (−1.38, 1.53)	0.23 (−1.22, 1.68)
VSD (%)	–4.97 (–9.43, –0.50)^*^	–4.79 (–9.24, –0.35)^*^	0.33 (0.06, 0.60)^*^	0.31 (0.04, 0.59)^*^	−0.92 (−3.59, 1.75)	−0.56 (−3.22, 2.10)	1.00 (−2.13, 4.13)	1.34 (−1.81, 4.49)
VDI (μm)	0.03 (0.01, 0.05)^*^	0.03 (0.01, 0.05)^*^	0.00 (0.00, 0.00)	0.00 (0.00, 0.00)	0.00 (−0.01, 0.01)	0.00 (−0.02, 0.01)	0.00 (−0.02, 0.01)	0.00 (−0.02, 0.01)
**OD**								
FAZ area (mm^2^)	0.15 (−0.07, 0.38)	0.16 (−0.06, 0.38)	0.01 (−0.01, 0.02)	0.01 (−0.01, 0.02)	0.01 (−0.12, 0.15)	0.02 (−0.11, 0.15)	−0.02 (−0.18, 0.14)	−0.02 (−0.18, 0.14)
FD-300 (%)	−0.69 (−2.02, 0.64)	−0.76 (−2.07, 0.55)	0.01 (−0.07, 0.09)	0.01 (−0.07, 0.09)	−0.03 (−0.82, 0.76)	−0.05 (−0.82, 0.73)	0.95 (0.00, 1.90)^*^	0.90 (−0.05, 1.84)
VD (%)	−0.66 (−2.67, 1.36)	−0.80 (−2.79, 1.18)	0.02 (−0.11, 0.14)	0.02 (−0.10, 0.14)	0.12 (−1.08, 1.31)	0.07 (−1.11, 1.25)	1.31 (−0.12, 2.75)	1.21 (−0.22, 2.64)
VSD (%)	−3.89 (−8.65, 0.86)	−4.69 (−9.40, 0.03)	0.08 (−0.21, 0.37)	0.09 (−0.21, 0.38)	0.60 (−2.23, 3.43)	0.50 (−2.32, 3.31)	1.35 (−1.99, 4.68)	0.93 (−2.42, 4.28)
VDI (μm)	0.01 (−0.01, 0.03)	0.01 (−0.01, 0.03)	0.00 (0.00, 0.00)	0.00 (0.00, 0.00)	0.00 (−0.01, 0.01)	0.00 (−0.01, 0.01)	0.01 (−0.01, 0.02)	0.01 (−0.01, 0.02)

OS, left eye; OD, right eye; FAZ, foveal avascular zone; FD-300, foveal vessel density in the 300 μm ring; VD, vessel density; VSD, vessel skeleton density; VDI, vessel diameter index; WMH, white matter hyperintensities; EPVS, enlarged perivascular spaces.

a Model 1 was adjusted for age, sex, education, and if applicable, for ICV; and Model 2 was additionally adjusted for dyslipidemia, diabetes, hypertension, smoking, and alcohol drinking.

* *P* < 0.05.

b Data on lacunes were missing in 6 participants.

### Associations of macular microvascular parameters with regional WMH volume

We further examined the associations of macular microvascular parameters with volumes of periventricular and deep WMH. Controlling for age, sex, education, and ICV, lower VSD and higher VDI of the left eye were significantly associated with larger PWMH volume (*P* < 0.05); the associations remained statistically significant even after further controlling for dyslipidemia, diabetes, hypertension, smoking, and alcohol consumption (*P* < 0.05; Table 3). In addition, lower VSD of the right eye was significantly associated with larger PWMH volume, even in the fully-adjusted model (*P* < 0.05; Table 3). There was no significant association between macular microvascular parameters and DWMH volume (*P* > 0.05; Table 3).

**Table 3:** β coefficient (95% confidence interval) of deep and periventricular white matter hyperintensity volume associated with macular microvascular parameters (*n* = 195).

**Macular microvascular index**	**Periventricular WMH volume (ml)**		**Deep WMH volume (ml)**	
	**Model 1^a^**	**Model 2^a^**	**Model 1^a^**	**Model 2^a^**
**OS**				
FAZ area (mm^2^)	0.02 (−0.17, 0.21)	0.04 (−0.16, 0.23)	−0.03 (−0.15, 0.08)	−0.02 (−0.13, 0.09)
FD-300 (%)	−0.52 (−1.79, 0.75)	−0.45 (−1.73, 0.83)	−0.46 (−1.21, 0.29)	−0.39 (−1.14, 0.36)
VD (%)	0.07 (−1.96, 2.10)	0.20 (−1.82, 2.21)	−0.51 (−1.71, 0.68)	−0.39 (−1.57, 0.78)
VSD (%)	–4.79 (–9.22, –0.37)^*^	–4.65 (–9.08, –0.22)^*^	−1.62 (−4.26, 1.02)	−1.52 (−4.13, 1.08)
VDI (um)	0.03 (0.01, 0.05)^*^	0.03 (0.01, 0.05)^**^	0.00 (−0.01, 0.02)	0.01 (−0.01, 0.02)
**OD**				
FAZ area (mm^2^)	0.18 (−0.04, 0.40)	0.19 (−0.03, 0.41)	−0.02 (−0.15, 0.12)	−0.01 (−0.14, 0.12)
FD-300 (%)	−0.49 (−1.81, 0.83)	−0.55 (−1.86, 0.75)	−0.61 (−1.38, 0.17)	−0.65 (−1.41, 0.11)
VD (%)	−0.36 (−2.35, 1.64)	−0.49 (−2.46, 1.49)	−0.82 (−2.00, 0.36)	−0.91 (−2.06, 0.24)
VSD (%)	−4.05 (−8.75, 0.65)	–4.76 (–9.45, –0.07)^*^	−0.28 (−3.09, 2.52)	−0.85 (−3.61, 1.92)
VDI (μm)	0.01 (−0.02, 0.07)	0.02 (−0.01, 0.04)	−0.01 (−0.02, 0.01)	−0.01 (−0.02, 0.01)

OS, left eye; OD, right eye; FAZ, foveal avascular zone; FD-300, foveal vessel density in the 300 μm ring; VD, vessel density; VSD, vessel skeleton density; VDI, vessel diameter index; WMH, white matter hyperintensities.

a Model 1 was adjusted for age, sex, education, if applicable, for ICV; Model 2 was additionally adjusted for dyslipidemia, diabetes, hypertension, smoking, and alcohol consumption.

* *P* < 0.05.

** P < 0.01.

### Interactions of macular microvascular parameters with sex on cerebral small vessel disease

**Figure 1:**
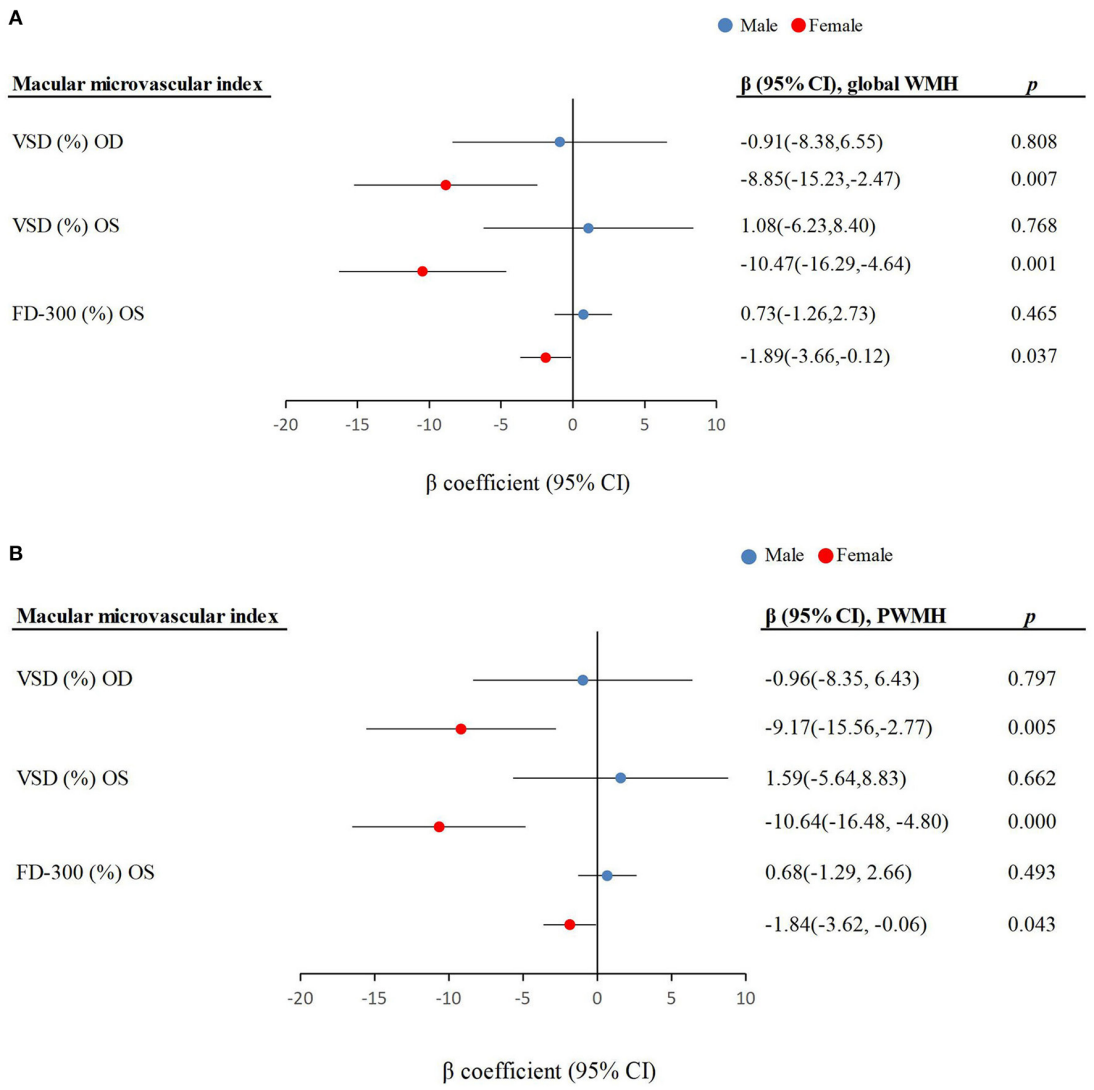
Associations of macular microvascular parameters with **(A)** global white matter hyperintensity volume and **(B)** periventricular white matter hyperintensity volume by sex (*n* = 195). CI, confidence interval; OD, right eye; OS, left eye; VSD, vessel skeleton density; FD-300, foveal vessel density in the 300 μm ring; PWMH, periventricular white matter hyperintensity.

We found statistical interactions of macular microvascular parameters (e.g., VSD and FD-300) with sex on the global WMH and PWMH volumes (*P*-interactions < 0.05). Further analyses stratified by sex showed that macular microvascular parameters (e.g., VSD and FD-300) were significantly associated with higher global WMH and PWMH volumes only among females not in males (*P* < 0.05; Figure 1).

## Discussion

In this population-based cross-sectional study of rural-dwelling Chinese older adults, we found that macular microvascular parameters (e.g., FAZ, FD-300, VSD, and VDI), especially in the left eye, were associated with signs of CSVD (e.g., WMH volume, EPVS count, and brain parenchymal volume). Furthermore, macular microvascular parameters (e.g., VSD and VDI) were associated with larger PWMH volume but not DWMH volume. Our study also revealed that the associations of macular vascular parameters (e.g., VSD and VDI) with volumes of global WMH and PWMH were only evident among females.

The associations between macular microvascular parameters and signs of CSVD have been reported in several case-control studies, but the findings were inconsistent (7, 25). The lower vascular density is indicative of reduced retinal blood flow. Our study indicated that lower vascular density (e.g., VSD and FD-300) was associated with higher global WMH volume and more EPVS, which were in line with the previous clinical-based studies (26, 27). Furthermore, we revealed that lower VSD and FD-300 were strongly associated with lower brain parenchymal volume, which has rarely been reported in previous studies. The FAZ area is most sensitive to ischemia and enlargement of FAZ area indicates macular ischemia (28). Our findings suggested that lower FAZ area was associated with more EPVS. However, a clinical-based case-control study in Shanghai found no association between FAZ area and EPVS count, which may be partially due to the fact that the case-control study included young adults (age ≥ 18 years) (26). VDI, which reflects vascular dilation, represents the average vessel caliber regardless of the vessel length (22). Our data suggested that higher VDI was associated with higher global WMH volume. This may hypothetically be due to the loss of smaller capillaries or compensatory vasodilation of the perfused capillaries secondary to a more hypoxic environment and increased local inflammatory molecules (29, 30). Previously, a population-based cohort study has linked the larger venular diameters with the progression of WMH (5). By contrast, the small-scale case-control study (*n* = 64) in Wuhan, China found no association between VDI and WMH (e.g., PWMH and DWMH), partly due to limited statistical power (31). Taken together, the findings from our population-based study and previous case-control studies suggest that macular microvascular parameters can be valuable markers for microvascular lesions in the brain.

PWMH and DWMH have distinct etiopathogenic mechanisms. PWMH is more likely to be determined by chronic hemodynamic insufficiency, whereas DWMH may be more attributed to small vessel disease (32). In line with this view, two hospital-based case-control studies suggested that lower vessel density was associated with severity of DWMH but not PWMH (26, 31). However, our study indicated that lower VSD and higher VDI were associated with higher PWMH volume, but not with DWMH volume. The discrepancy across studies may be partly attributed to differences in the study settings (e.g., clinical vs. the general population setting) and characteristics of study populations (e.g., young adults vs. older adults). Thus, further longitudinal studies with larger sample sizes are needed to clarify the potential causal relationship between macular microvascular parameters and WMH by anatomic locations.

In this study, we further examined the interactions of macular microvascular parameters with sex on CSVD markers and found that the associations of macular vascular parameters (e.g., VSD and VDI) with volumes of global WMH and PWMH were only evident among females. This was consistent with the previous community-based study, which showed that associations between WMH severity and arteriovenous nicking were stronger in women than in men (33). It has been hypothesized that estrogens can increase tissue perfusion of the retina and brain probably by reducing vascular resistance, protecting against oxidative stress, and stimulating synaptogenesis in premenopausal women (34, 35). However, estrogen levels decline markedly after the menopause, the vascular protection of estrogen disappears and women become more susceptible to vascular changes and diseases in the brain (34, 36).

Another interesting observation from our study is that the associations between macular microvascular parameters and CSVD were evident mainly in the left eye. This is in agreement with a hospital-based study of adults (age ≥ 18 years) in Beijing, which showed that lower VD of the left eye, but not right eye, was associated with higher burdens of WMH and EPVS (37). This may partially be attributed to structural and functional brain asymmetry occurred during normal human brain development (32, 38). Furthermore, previous studies also showed that macular microvascular signs of left eye reflect function and connectivity of the right hemisphere (39) and the small vascular lesions of the right hemisphere occur earlier and are much more severer than those of the left hemisphere (40). Finally, there was evidence showing that the left eye appeared to be more sensitive to damage due to hypoxia compared with the right eye (41).

The pathophysiologic mechanisms underlying the associations of macular microvascular parameters with CSVD are not fully understood but can be speculated. First, anatomically, the retina is regarded as an extension of the diencephalon and has a similar pattern of angiogenesis. Physiologically, similar to the brain, the retina has a highly isolated and protected vascular system (3, 42). Second, retinal and brain microvascular lesions share common cardiovascular risk factors (43). Third, dysfunction of the blood-retina barrier from retina hypoxia owing to long-term exposures to cardiovascular risk factors, which is analogous to and associated with the blood-brain barrier dysfunction, might play a role in the pathogenesis of both retinal microvascular changes and CSVD (42, 44). Finally, inflammation and endothelial dysfunction may also be involved in the process of retinal and cerebral microangiopathy (29). Given the homology of microvasculature in the brain and the retina, the link of macular microvascular parameters with signs of CSVD may reflect underlying pathological processes common in both the brain and the retina (25, 27).

The major strengths of our study include the population-based design and comprehensive assessments of macular microvascular and CSVD indicators. Our study also has limitations. First, the cross-sectional study cannot determine a causal relationship. Second, the study sample was relatively small and the statistical power might not be big enough to detect weak-to-moderately strong associations between retinal parameters and markers of CSVD. Third, data on cerebral microbleeds and microinfarcts were not available, which might underestimate the associations of macular microvascular parameters with CSVD. Finally, the study participants were derived from just one rural area and they were also slightly younger and more educated than the MIND-China total sample, which might not be representative of rural population in China. These should be borne in mind when extrapolating the findings to other rural populations.

## Conclusions

In summary, our population-based study of rural-dwelling older adults showed that macular microvascular parameters were independently associated with CSVD markers, and that the observed associations with WMH volume were evident mainly among females. This suggests that quantitative macular microvascular parameters could be useful markers for CSVD. These findings may have potential implications for clinical management of macular microvascular signs in older adults. For instance, ophthalmologists should be aware of the potential link of macular microvascular signs with brain lesions. Structural brain MRI examination may be considered when abnormal macular microvascular signs were detected, especially among older women. Future large-scale prospective cohort studies are warranted to clarify the potential causal relationships of macular microvascular signs with CSVD markers as well as the mechanisms underlying their associations. In addition, the functional consequences (e.g., cognitive and physical dysfunction) of macular microvascular signs among older adults deserve further investigation.

## Data availability statement

The raw data supporting the conclusions of this article will be made available by the authors, without undue reservation.

## Ethics statement

The studies involving human participants were reviewed and approved by Ethics Committee at Shandong Provincial Hospital. The patients/participants provided their written informed consent to participate in this study.

## Author contributions

ZX, QZ, YDu, and CQ: study concept and design. ZX, YW, LS, SN, SW, MZ, JW, YDo, TH, LC, ST, and XH: data collection and assessments. ZX: data analysis and writing the first draft of the manuscript. YDu and CQ: study supervision. All authors have critically revised the manuscript for important intellectual content and approved the final manuscript.
